# 

**Published:** 1881-09

**Authors:** 


VOL. II. SEPTEMBER, 1881. NO. 9.

— THE —

### Independent Practitioner:

A MONTHLY JOURNAL,

DEVOTED TO

### Medical, Surgical, Obstetrical, Dental, Hygienical and Popular Sciences.

S.G.O.L. INDEXED

EDITED BY
HARVEY L. BYRD, A. M., M. D.,
AND
BASIL M. WILKERSON, D. D. S., M. D.

**"Altera poscit opem res, et conjurat amice."**

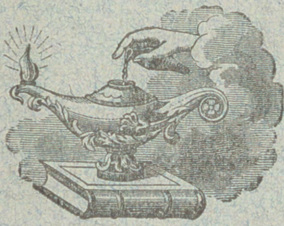

NEW YORK:
*Published by B. M. WILKERSON, Proprietor,*
Office, 20 Courtlandt Street.
Wm. Miller, Business Manager.

$3.00 Per Annum in Advance. - - Single Copies 30 Cents.

Entered at the Post-Office, New York City, as Second-class matter.

